# Stat3 signaling regulates embryonic stem cell fate in a dose-dependent manner

**DOI:** 10.1242/bio.20149514

**Published:** 2014-09-19

**Authors:** Chih-I Tai, Eric N. Schulze, Qi-Long Ying

**Affiliations:** Eli and Edythe Broad Center for Regenerative Medicine and Stem Cell Research at USC, Department of Stem Cell Biology and Regenerative Medicine, Keck School of Medicine, University of Southern California, Los Angeles, CA 90033, USA; * Present address: Animal Biotechnology Interdisciplinary Group, Center for Veterinary Medicine, United States Food and Drug Administration, 7500 Standish Place, Rockville, MD 20855, USA.

**Keywords:** Stat3, Embryonic stem cell, Pluripotency

## Abstract

Stat3 is essential for mouse embryonic stem cell (mESC) self-renewal mediated by LIF/gp130 receptor signaling. Current understanding of Stat3-mediated ESC self-renewal mechanisms is very limited, and has heretofore been dominated by the view that Stat3 signaling functions in a binary “on/off” manner. Here, in contrast to this binary viewpoint, we demonstrate a contextual, rheostat-like mechanism for Stat3's function in mESCs. Activation and expression levels determine whether Stat3 functions in a self-renewal or a differentiation role in mESCs. We also show that Stat3 induces rapid differentiation of mESCs toward the trophectoderm (TE) lineage when its activation level exceeds certain thresholds. Stat3 induces this differentiation phenotype via induction of *Tfap2c* and its downstream target *Cdx2*. Our findings provide a novel concept in the realm of Stat3, self-renewal signaling, and pluripotent stem cell biology. Ultimately, this finding may facilitate the development of conditions for the establishment of authentic non-rodent ESCs.

## INTRODUCTION

Embryonic stem cells (ESCs) were originally derived by explanting mouse blastocysts onto a layer of mitotically inactivated fibroblasts (‘feeders’) in medium containing fetal calf serum (FCS) (Evans and Kaufman, 1981; Martin, 1981). Under this condition, mouse ESC (mESC) lines can be derived and subsequently propagated indefinitely. Leukemia inhibitory factor (LIF), an interleukin-6 (IL-6) family member, can replace feeder cells in serum-containing medium for derivation of ESCs from the 129 strain of mice (Smith et al., 1988; Williams et al., 1988). LIF maintains mESC self-renewal through activation of the signal transducer and activator of transcription 3 (Stat3). Intriguingly, strains of mice vary greatly in the ease with which their embryos can be directed to give rise to ESC lines under the LIF+FCS condition (Kawase et al., 1994), and the underlying mechanism remains largely unknown. The 129 strain has been found to be the most permissive for ESC derivation (Brook and Gardner, 1997). 129 mESCs can be derived and maintained in the absence of feeders in medium supplemented with LIF and FCS (Nagy et al., 1993). It has been observed that 129 strains harbor a genetic predisposition to a high testicular germ cell tumor formation frequency. This increased tumor risk is hypothesized to account for, in part, the significantly higher ability to form pluripotent embryonal carcimona (EC) cell lines and ESC lines in this mouse strain (Brook and Gardner, 1997). To date, no ESC lines have been derived from any non-129 strains of mice in the LIF+FCS condition without the use of feeders. Given the difficulty and high failure rate of most attempts to derive non-129 mESC lines, we sought to investigate whether the difference in derivation efficiency, could in part, be explained by previously undescribed dose-dependent effect of Stat3 in the regulation of ESC self-renewal.

Once LIF binds to the LIF receptor (LIFR), the receptor forms a heterodimer with the membrane protein, gp130. The heterodimerization of LIFR and gp130 triggers the membrane-proximal cytosolic docking and activation of the associated Janus kinases (JAKs) (Lütticken et al., 1994; Stahl et al., 1994). The activated JAKs phosphorylate the tyrosine residues on gp130 to create a docking site for Stat3 recruitment and in turn, activate Stat3 by phosphorylating its tyrosine 705 residue (Boeuf et al., 1997; Darnell, 1997). Activated Stat3s form homodimers and subsequently translocates into the nucleus to regulate gene transcription. Stat3 phosphorylation at tyrosine 705 is essential for mESC-self-renewal mediated by Stat3 (Evans and Kaufman, 1981; Huang et al., 2014; Niwa et al., 1998). Artificial activation of Stat3, bypassing LIF and LIF/gp130 receptors altogether, can maintain mESCs in an undifferentiated state (Matsuda et al., 1999). LIF/Stat3 signaling activates multiple downstream targets in mESCs. LIF/Stat3-mediated mESC self-renewal can be partially recapitulated by overexpressing some of these downstream targets, including Tfcp2l1, Gbx2, Klf4, Klf5, Pim1, Pim3, Pramel7, and c-Myc (Aksoy et al., 2007; Cartwright et al., 2005; Casanova et al., 2011; Li et al., 2005; Martello et al., 2013; Parisi et al., 2008; Tai and Ying, 2013; Ye et al., 2013).

Current understanding of Stat3-mediated mESC self-renewal has been dominated by the view that LIF/Stat3 signaling functions in a binary “on/off” manner. It has not been clear whether Stat3 activation level is critical to ESC self-renewal and whether non-129 strain mESCs can be maintained under feeder-free conditions through modulation of Stat3 activity. Here, we use chemical and genetic approaches to modulate Stat3 activity in mESCs and determined the corresponding effect on self-renewal and differentiation. We report that Stat3 exhibits a dose-dependent effect in mESC self-renewal and differentiation.

## RESULTS

### Enhancing Stat3 activity liberates derivation-refractory mESCs from the dependence of feeders

**Fig. 1.**
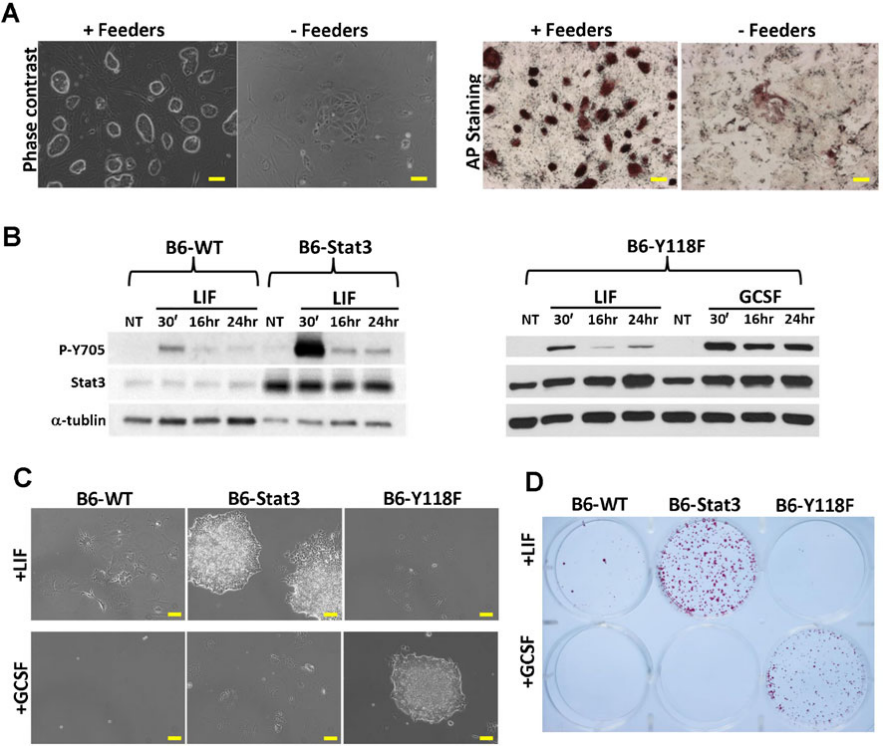
Enhancing Stat3 activity liberates B6 ESCs from the dependence of feeders. (A) Phase contrast (left panel) and AP staining images (right panel) of B6 ESCs cultured in mESC medium supplemented with LIF with or without feeders for 7 days. (B) Western blot analysis of phospho-Stat3 (Tyr705) and total Stat3levels in wild-type B6 (B6-WT), B6-Stat3 (left panel) and B6-Y118F mESCs (right panel) treated with LIF or GCSF for the indicated times. NT: no treatment. (C) Phase contrast images of B6-WT, B6-Stat3, and B6-Y118F mESCs cultured in mESC medium supplemented with either LIF or GCSF in the absence of feeders for 7 days. ESCs were plated into 0.1% gelatin-coated 6-well plates at a density of 1000 cells/well. (D) AP staining result of panel C. Scale bars: 50 µm.

B6 mESCs were derived from the C57BL/6 strain of mouse and are routinely maintained by co-culturing with feeders in the presence of LIF and FCS (Ye et al., 2012) When removed from feeders and cultured in mESC medium supplemented with LIF, these B6 mESCs died or differentiated and could not be continuously propagated (Fig. 1A). We examined total and phosphorylated Stat3 levels in LIF-stimulated B6 mESCs and found that they were both markedly lower than that of LIF-stimulated 46C mESCs, a feeder-independent ESC line derived from the 129 strain of mouse (supplementary material Fig. S1A,B) (Ying et al., 2003). Therefore, we asked whether enhancing Stat3 activity is sufficient to maintain B6 mESC self-renewal under feeder-free conditions. We introduced a Stat3 transgene into B6 mESCs to increase overall Stat3 expression (hereafter termed “B6-Stat3”). We also engineered B6 mESCs to express a gp130-Y118F chimeric receptor (B6-Y118F) which consists of the extracellular domain of the granulocyte colony-stimulating factor (GCSF) receptor fused to the transmembrane and cytoplasmic region of gp130 containing a phenylalanine to tyrosine substitution at residue 118 (Y118F). This Y118F mutation blocks the binding of suppressor of cytokine signaling 3 (Socs3) to gp130 receptor, and therefore prevents the negative feedback loop mediated by Socs3 (Burdon et al., 1999). As a result, Stat3 can be activated by GCSF in a dose-dependent manner, bypassing the endogenous LIF/gp130 receptors which might be limited in ESCs. As expected, increased total and phosphorylated Stat3 levels were observed in B6-Stat3 mESCs compared to B6 mESCs. Phosphorylated Stat3 levels in B6-Y118F mESCs treated with GCSF were also significantly higher and sustained much longer than that of LIF-treated B6 mESCs (Fig. 1B), likely because Stat3 phosphorylation through gp130-Y118F chimeric receptor can bypass the Socs3-mediated negative feedback loop (Burdon et al., 1999; Hirano et al., 2000). Both B6-Stat3 and B6-Y118F mESCs could be maintained without feeders in the presence of LIF and GCSF, respectively (Fig. 1C,D), suggesting that enhancing Stat3 activity can liberate B6 mESCs from the dependence of feeders.

### Stat3 induces differentiation of mESCs when its activation level exceeds certain thresholds

**Fig. 2.**
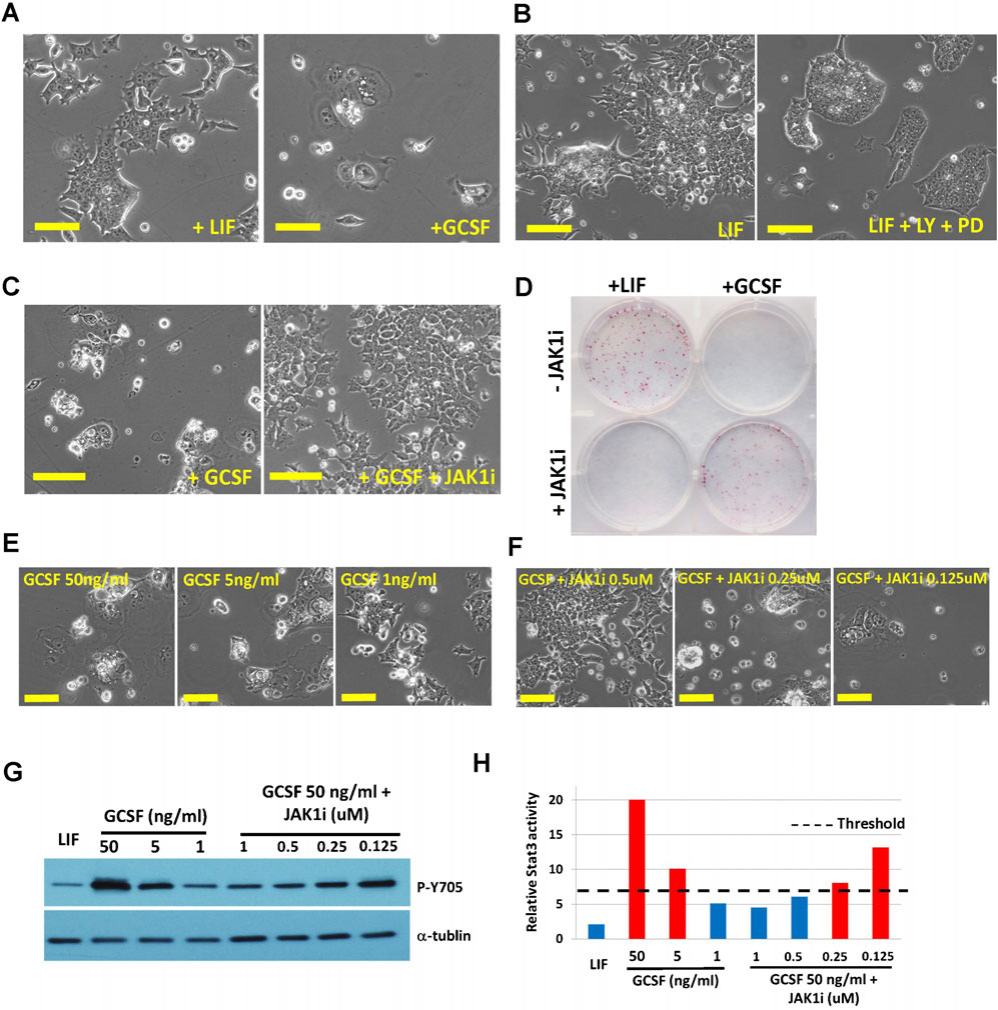
Hyperactivation of Stat3 induces ESC differentiation. (A) Phase contrast images of B6-S3Y118F mESCs cultured in mESC medium supplemented with LIF (left panel) or GCSF for 24 hours (right panel). (B) Phase contrast images of B6-S3Y118F mESCs cultured in the presence of LIF (left panel), or LIF plus 5 µM LY294002 (PI3K inhibitor) and 1 µM PD0325901 (MEK inhibitor) (right panel) for 48 hours. (C) Phase contrast images of B6-S3Y118F mESCs cultured in the presence of GCSF, or GCSF plus 1 µg/ml JAK1i for 48 hours. (D) AP staining of B6-S3Y118F mESCs cultured in the presence of LIF or GCSF with or without JAK1i for 7 days. (E) Phase contrast images of B6-S3Y118F mESCs cultured in the presence of various doses of GCSF for 3 days. (F) Phase contrast images of B6-S3Y118F mESCs cultured in the presence of 50 ng/ml GCSF plus various doses of JAK1i for 3 days. (G) Western blot analysis of phospho-Stat3 (Tyr705) levels of B6-S3Y118F mESCs treated with different doses of GCSF and JAK1i for 24 hours. (H) Quantification of the normalized phospho-Stat3 (Tyr705) levels against endogenous α-tublin from the result in panel G. Red bar indicates the conditions that mESCs undergo differentiation. Blue bar indicates the conditions that mESCs can be maintained undifferentiated. Scale bars: 50 µm.

To further define how Stat3 activation level can affect ESC fate, we introduced both Stat3 and gp130-Y118F transgenes into B6 mESCs (hereafter called B6-S3Y118F mESCs) so that we could broadly manipulate Stat3 activation level. B6-S3Y118F mESCs remained undifferentiated without feeders in the presence of LIF (Fig. 2A, left panel). Surprisingly, B6-S3Y118F mESCs differentiated after administration of 50 ng/ml GCSF for 24 hours (Fig. 2A, right panel). LIF activates JAK/Stat3 as well as PI3K/AKT and MEK/ERK signaling pathways, while GCSF activates JAK/Stat3 but not PI3K/AKT and MEK/ERK pathways in B6-S3Y118F mESCs due to the lack of the Src homology phosphatase 2 (SHP2) docking site in the chimeric gp130-Y118F chimeric receptor (Burdon et al., 1999). In effect, only Stat3 signaling is activated by GCSF in cells expressing the chimeric receptor. To determine whether lack of PI3K/AKT or/and MEK/ERK activation is responsible for the differentiation phenotype, we treated the cells with chemical inhibitors of these two pathways (LY294002 and PD0325901) prior to the administration of LIF to the cells. We found that B6-S3Y118F mESCs remained undifferentiated after administration of the inhibitors along with LIF (Fig. 2B), suggesting that lack of PI3K/AKT or MEK/ERK signaling is unlikely the cause of the rapid differentiation phenotype. Next, we asked whether the phenotype was correlated with Stat3 activation levels. JAK1 activity is required for Stat3 activation through regulation of Stat3 phosphorylation (Ernst et al., 1996), therefore, we used a selective JAK1 inhibitor (JAK1i) to attenuate Stat3 activity in mESCs. Indeed, GCSF-induced differentiation in B6-S3Y118F mESCs could be prevented by co-administration of 1 µg/ml JAK1i (Fig. 2C,D). Contrarily, B6-S3Y118F mESCs could not be maintained in LIF plus 1 µg/ml JAK1i (Fig. 2D). Taken together, these results suggest that excess Stat3 activation is the central cause of B6-S3Y118F mESC differentiation induced by GCSF.

To further confirm that Stat3 activity is correlated with the rapid differentiation phenotype, we modulated Stat3 activity by applying different concentrations of GCSF and JAK1i. Indeed, B6-S3Y118F mESCs self-renewal could be maintained at a lower concentration of GCSF or at a higher concentration of GCSF co-administered with an adequate dosage of JAK1i (Fig. 2E,F). We examined Stat3 phosphorylation levels in B6-S3Y118F mESCs and found that the Stat3 phosphorylation level was significantly elevated in B6-S3Y118F mESCs treated with GCSF than ESCs treated with LIF and that the Stat3 phosphorylation level could be modulated via applying varying concentrations of GCSF and JAKi. (Fig. 2G). By performing quantitative analysis of Stat3 phosphorylation levels and correlating them to ESC self-renewal and differentiation phenotypes, we were able to determine a threshold that distinguishes whether Stat3 activation induces ESC differentiation or self-renewal (Fig. 2H). Once this threshold is exceeded, activation of Stat3 switches its role in self-renewal to that of inducing differentiation.

To determine whether hyperactivated Stat3 inducing rapid differentiation is a general phenomenon in mESCs, we introduced Stat3 and chimeric gp130-Y118F transgenes into E14 mESCs, a widely-used feeder-independent ESC line derived from the 129 strain of mouse. Both E14-Y118F and E14-S3Y118F mESCs could be maintained in LIF condition (supplementary material Fig. S2A). However, when treated with 50 ng/ml of GCSF, E14-S3Y118F ESCs underwent rapid differentiation whereas E14-Y118F ESCs remained undifferentiated (supplementary material Fig. S2A). Western blot analysis confirmed that the level of Stat3 phosphorylation in E14-S3Y118F mESCs treated with GCSF for 6 hours was significantly higher than those treated with LIF (supplementary material Fig. S2B,C). The differentiation phenotype of E14-S3Y118F mESCs induced by GCSF could also be rescued by co-administration of JAK1i (supplementary material Fig. S2D).

### Hyperactivation of Stat3 in ESCs promotes trophectoderm differentiation

**Fig. 3.**
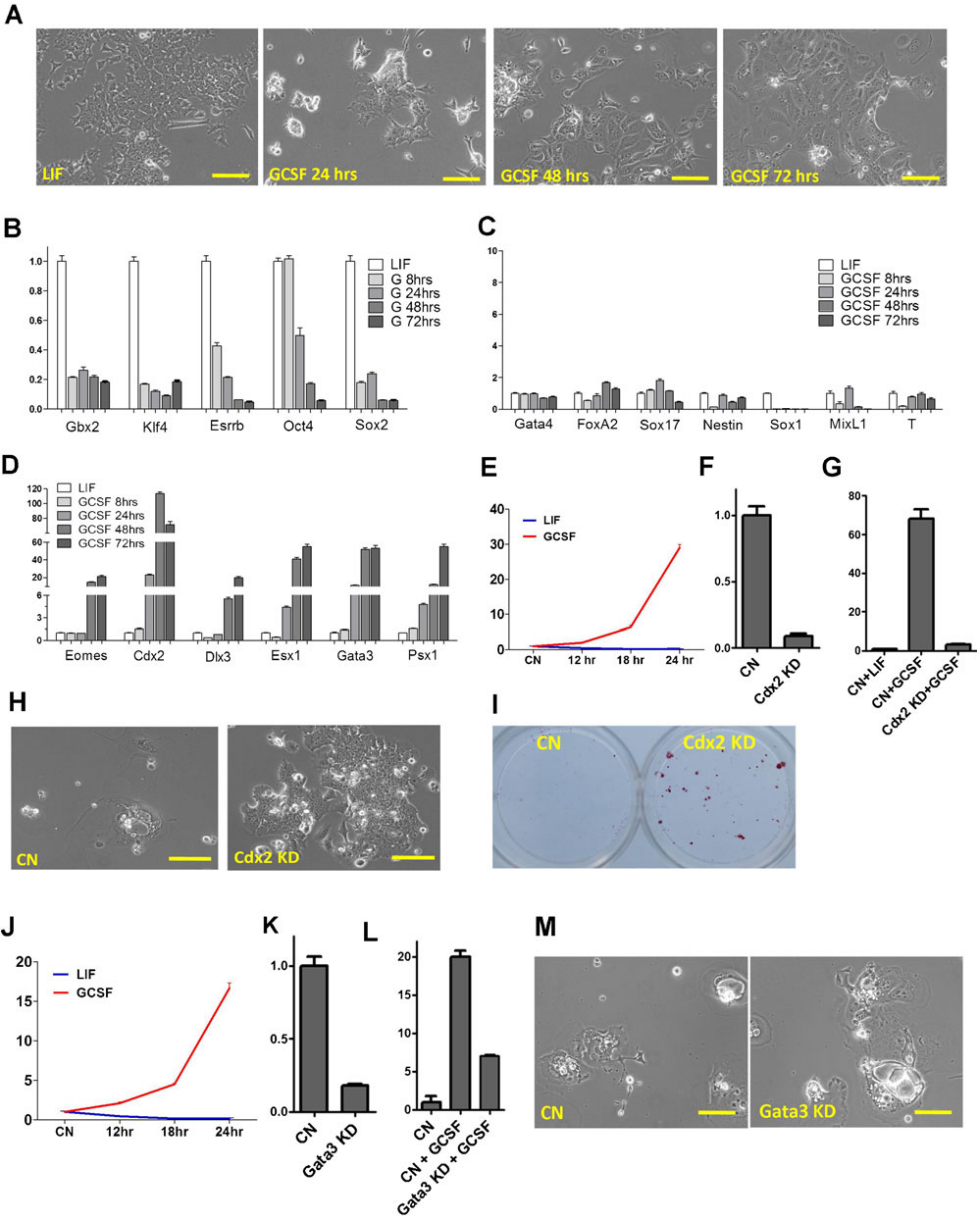
Cdx2 knockdown blocks TE differentiation of ESCs induced by Stat3 hyperactivation. (A) Phase contrast images of B6-S3Y118F mESCs maintained in LIF or treated with GCSF for the indicated times. (B) qPCR analysis of the expression of pluripotency genes in B6-S3Y118F mESCs maintained in LIF or treated with GCSF for the indicated times. (C) qPCR analysis of gene expression in B6-S3Y118F mESCs maintained in LIF or treated with GCSF for the indicated times. Endoderm markers: Gata4, FoxA2, Sox17; ectoderm markers: Nestin, Sox1; mesoderm markers: MixL1, T. (D) qPCR analysis of gene expression in B6-S3Y118F mESCs maintained in LIF or treated with GCSF for the indicated times. Eomes, Cdx2, Dlx3, Esx1, Gata3, and Psx1 are markers of TE. (E) qPCR analysis of *Cdx2* expression in B6-S3Y118F mESCs treated with GCSF or LIF for the indicated times. B6-S3Y118F mESCs were starved overnight in serum free medium prior to the treatments. (F) qRT-PCR analysis of *Cdx2* expression in B6-S3Y118F mESCs expressing scramble or *Cdx2* shRNAs. (G) qRT-PCR analysis of *Cdx2* expression in scramble or *Cdx2* shRNAs-expressing B6-S3Y118F mESCs treated with LIF or GCSF for 24 hours. (H) Phase contract images of scramble (CN) or *Cdx2* shRNAs-expressing B6-S3Y118F mESCs cultured in the presence of GCSF for 7 days. (I) AP staining of scramble or *Cdx2* shRNAs-expressing B6-S3Y118F mESCs cultured in the presence of GCSF for 7 days. (J) qPCR analysis of *Gata3* expression in B6-S3Y118F mESCs treated with GCSF or LIF for the indicated times. B6-S3Y118F mESCs were starved overnight in serum free medium prior to the treatments. (K) qRT-PCR analysis of *Gata3* expression in scramble or *Gata3* shRNAs-expressing B6-S3Y118F mESCs maintained in LIF. (L) qRT-PCR analysis of *Gata3* expression in scramble or *Gata3* shRNAs-expressing B6-S3Y118F mESCs treated with GCSF for 24 hours. (M) Phase contract images of scramble (CN) or *Gata3* shRNAs-expressing B6-S3Y118F mESCs cultured in the presence of GCSF for 7 days. Scale bars: 50 µm. Error bars represent the s.d. (n = 4).

B6-S3Y118F mESCs underwent rapid differentiation when Stat3 was hyperactivated via GCSF-mediated Stat3 activation (Fig. 3A). To determine which cell lineages are induced by Stat3 hyperactivation, we performed RT-PCR analysis of gene expression in B6-S3Y118F mESCs treated with GCSF at different time points. Expression of ESC markers, *Gbx2*, *Klf4*, *Oct4*, *Sox2*, and *Esrrb* was significantly downregulated after treatment with 50 ng/ml GCSF for 24 hours (Fig. 3B), confirming that ESC underwent rapid differentiation after GCSF treatment. Not surprisingly, expression of *Gbx2* and *Klf4*, which are also the downstream targets of Stat3, were initially upregulated but then quickly downregulated 4–8 hours after the addition of GCSF (supplementary material Fig. S3), indicating rapid differentiation of B6-S3Y118F mESCs induced by GCSF.

Next, we examined the expression levels of various germ layer markers in B6-S3Y118F mESCs treated with GCSF. Surprisingly, GCSF treatment did not significantly upregulate any of the three somatic germ layer markers examined (Fig. 3C); instead, it specifically induced the expression of trophectoderm (TE) markers (Fig. 3D; supplementary material Fig. S4). Additionally, the differentiated cells induced by GCSF cannot be maintained or passaged in the trophoblast stem cell culture condition, suggesting that GCSF induces B6-S3Y118F mESC differentiation towards terminally differentiated TE cells (supplementary material Fig. S5).

### Cdx2 and Tfap2c are the two key factors that mediate TE differentiation of ESCs induced by Stat3 hyperactivation

Among the TE markers, *Cdx2* and *Gata3* were significantly upregulated within 24 hours of GCSF treatment in B6-S3Y118F mESCs (Fig. 3D). Notably, both Cdx2 and Gata3 have been reported to be the key regulators in TE differentiation and overexpressing either one is sufficient to induce mESC differentiation towards the TE lineage (Niwa et al., 2005; Ralston et al., 2010; Ray et al., 2009; Strumpf et al., 2005). Therefore, we asked whether early induction of Cdx2 or Gata3 contributes to TE differentiation triggered by Stat3 hyperactivation. We first examined the *Cdx2* expression profile in B6-S3Y118F mESCs treated with GCSF or LIF and found that *Cdx2* expression was induced by GCSF but not by LIF (Fig. 3E). shRNA-mediated knockdown of *Cdx2* could prevent TE differentiation of B6-S3Y118F mESCs induced by GCSF (Fig. 3F–I). *Gata3*, likes *Cdx2*, was also induced by GCSF but not by LIF in B6-S3Y118F mESCs (Fig. 3J). shRNA-mediated *Gata3* knockdown (Fig. 3K,L), however, was not sufficient to block TE differentiation induced by GCSF (Fig. 3M). Together, these results suggest that Cdx2 mediates TE differentiation of mESCs induced by Stat3 hyperactivation.

**Fig. 4.**
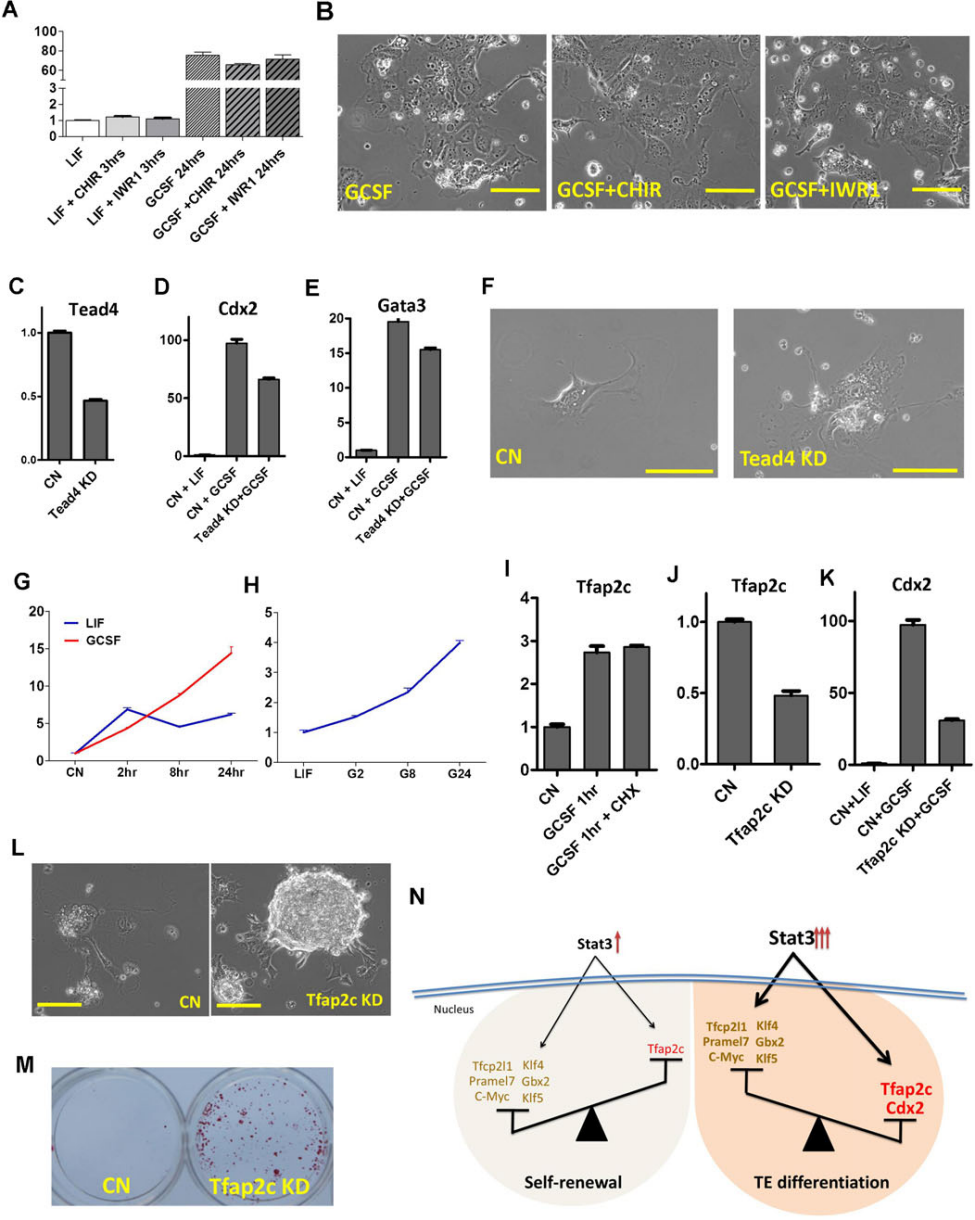
Tfap2c, a direct target of Stat3, cooperates with MAPK pathway to induce Cdx2 expression. (A) qRT-PCR analysis of *Cdx2* expression in B6-S3Y118F mESCs cultured in the indicated conditions. (B) Phase contrast images of B6-S3Y118F mESCs cultured in the indicated conditions for 5 days. (C) qRT-PCR analysis of *Tead4* expression in scramble or *Tead4* shRNAs-expressing B6-S3Y118F mESCs. (D) qRT-PCR expression of *Cdx2* expression in scramble or *Tead4* shRNAs-expressing B6-S3Y118F mESCs treated with GCSF for 24 hours. (E) qRT-PCR analysis of *Gata3* expression in scramble or *Tead4* shRNAs-expressing B6-S3Y118F mESCs treated with GCSF for 24 hours. (F) Phase contrast images of scramble (CN) or *Tead4* shRNAs-expressing B6-S3Y118F mESCs cultured in the presence of GCSF for 7 days. (G) qPCR analysis of *Tfap2c* expression in B6-S3Y118F mESCs treated with GCSF or LIF for the indicated times. B6-S3Y118F mESCs were starved overnight in serum free medium prior to the treatments. (H) qPCR analysis of *Tfap2c* expression in B6-S3Y118F mESCs treated with GCSF for the indicated times. B6-S3Y118F mESCs were maintained in the LIF condition prior to GCSF treatment. (I) qPCR analysis of *Tfap2c* expression in B6-S3Y118F mESCs treated with GCSF or GCSF plus cycloheximide (CHX) for 1 hour. B6-S3Y118F mESCs were starved overnight in serum free medium prior to the treatment. (J) qRT-PCR analysis of *Tfap2c* expression in scramble or *Tfap2c* shRNAs-expressing B6-S3Y118F mESCs. (K) qRT-PCR analysis of *Cdx2* expression in scramble or *Tfap2c* shRNAs-expressing B6-S3Y118F mESCs treated with GCSF for 24 hours. (L) Phase contrast images of scramble (CN) or *Tfap2c* shRNAs-expressing B6-S3Y118F mESCs cultured in the presence of GCSF for 7 days. (M) AP staining of scramble or *Tfap2c* shRNAs-expressing B6-S3Y118F mESCs cultured in the presence of GCSF for 7 days. (N) Model of mESC fate regulated by Stat3 activation level. Scale bars: 50 µm. Error bars represent the s.d. (n = 4).

Cdx2 expression has been shown to be regulated by WNT/β-catenin (Chen et al., 2013) and Hippo/Tead4 pathways (Yagi et al., 2007). We asked next whether these two pathways are involved in Stat3 hyperactivation-mediated Cdx2 induction. We applied previously characterized small molecules to activate or inhibit WNT/β-catenin signaling and found no significant changes in *Cdx2* expression following GCSF treatment in B6-S3Y118F mESCs (Fig. 4A), nor did these small molecules prevent GCSF-induced TE differentiation (Fig. 4B). Knockdown of *Tead4* partially downregulated the expression of *Cdx2* and *Gata3* in B6-S3Y118F mESCs treated with GCSF (Fig. 4C–E). However, *Tead4* knockdown did not prevent the TE differentiation (Fig. 4F). These results imply that neither WNT/β-catenin nor Hippo/TEAD4 signaling is a significant regulator of Cdx2 induction caused by Stat3 hyperactivation.

Among the Stat3 direct targets we identified through microarray analysis (GSE38719), we found that *Tfap2c* is a potential candidate linking Stat3 to TE differentiation. Tfap2c is required for embryonic development and proliferation of extraembryonic TE cells (Werling and Schorle, 2002). Forced expression of Tfap2c promotes TE differentiation in mESC through associating with Cdx2 or its regulatory-binding loci (Adachi et al., 2013; Kuckenberg et al., 2010). We observed that *Tfap2c* expression was continuously upregulated in B6-S3Y118F mESCs treated with GCSF, whereas LIF-induced expression of *Tfap2c* peaks and then plateaus within 2 hours of stimulation (Fig. 4G). To further validate that GCSF induces an increased *Tfap2c* expression compared to LIF, we examined *Tfap2c* expression by sequentially giving GCSF to B6-S3Y118F mESCs maintained in LIF. Similarly, GCSF treatment can exceed the limit of basal *Tfap2c* expression induced by LIF and continuously induce its expression (Fig. 4H). We next used a *de novo* protein synthesis inhibitor, cycloheximide (CHX), to verify whether *Tfap2c* is directly regulated by Stat3. Despite CHX application, *Tfap2c* expression level increased significantly following a one-hour GCSF application, suggesting that GCSF directly regulates *Tfap2c* expression (Fig. 4I). Additionally, a previous study has indicated an association between Stat3 and *Tfap2c* loci (Chen et al., 2008). Taken together, these results suggest that *Tfap2c* is a direct downstream target of Stat3 and that Stat3 hyperactivation can sustain *Tfap2c* induction.

To determine whether Stat3 hyperactivation induces TE differentiation through upregulation of Tfap2c, we knocked down *Tfap2c* expression by shRNA (Fig. 4J). *Tfap2c* knockdown reduced GCSF-mediated *Cdx2* induction (Fig. 4K) and prevented TE differentiation (Fig. 4L,M). Our results suggest that Tfap2c and Cdx2 are the two key factors that mediate TE differentiation of mESCs induced by Stat3 hyperactivation.

## DISCUSSION

In this study, we describe a previously unknown function for Stat3 in mESCs, specifically, that Stat3's self-renewal function is contextual and dose-dependent. We also address a long-standing technical and practical question regarding mESC culturing, that is, how to promote feeder-free self-renewal in non-permissive (derivation-refractory) mESC strains through modulation of Stat3 activity. LIF-induced Stat3 activity is not sufficient to maintain non-129 ESC self-renewal under feeder-free conditions, and enhancing Stat3 activity through overexpression of a Stat3 transgene or a gp130 chimeric receptor is necessary and sufficient to obviate the requirement of the feeder layer and maintain an undifferentiated ESC state. However, elevating Stat3 activity over a certain threshold will switch Stat3's self-renewal promoting effect into one that induces ESC differentiation towards the TE lineage.

How does Stat3 play such contradictory roles in the same pluripotent stem cells? Upon stimulation by cytokines, Stat3 is first recruited to the receptors via its Src-homology-2 (SH2) domain, and then phosphorylated on tyrosine 705, leading to dimerization and translocation to the nucleus, where it binds specific DNA sequences and activates target gene transcription. We hypothesize that Stat3 recruits distinct co-activators and activates distinct gene programs depending on its activation level. Stat3 can not only regulate the expression of pluripotency-related genes such as *Tfcp2l1*, *Gbx2*, *Klf4*, *Klf5*, *Pim1*, *Pim3*, *Pramel7*, and *c-Myc* (Aksoy et al., 2007; Cartwright et al., 2005; Casanova et al., 2011; Li et al., 2005; Martello et al., 2013; Parisi et al., 2008; Tai and Ying, 2013; Ye et al., 2013), but also induces the expression of factors that promote TE differentiation (Fig. 4N). The outcome of Stat3 activation in mESCs, whether to promote self-renewal or induce differentiation, is likely determined by the balance of expression level between pluripotency- and differentiation-related genes both induced by Stat3 (Fig. 4N). When Stat3 is activated by LIF, Tfap2c expression can be temporarily induced but remains constantly low (Fig. 4G). The pluripotency factors parallelly induced by Stat3 then promote self-renewal of mESCs. However, when Stat3 is highly and sustainably activated by GCSF in B6-S3Y118F mESCs, the expression of Tfap2c can be continuously induced and consequently upregulates Cdx2 expression. Both Tfap2c and Cdx2 can override the self-renewal-promoting effect of the pluripotency genes induced by Stat3 and induce mESC differentiation towards the TE lineage (Fig. 4N) (Adachi et al., 2013; Kuckenberg et al., 2010).

What is the molecular mechanism underlying Stat3's dose-dependent effect in regulating ESC fates? One possible mechanism is that the binding of Stat3 with different sets of co-factors may be determined by its activation level. When Stat3 is normally activated, Stat3 may favor to bind to cofactors that promote self-renewal than to cofactors that induce differentiation. On the other hand, when Stat3 is hyperactivated, more activated Stat3 translocates into nucleus than when it is normally activated. Stat3 will then first occupy cofactors that promote self-renewal and the excess Stat3 then starts to bind to a different set of cofactors that induce differentiation. The expression of the differentiation-inducing genes will then override the self-renewal promoting genes and lead ESCs to differentiation. This hypothesis explains why, in mESCs, Stat3 has to be hyperactivated to induce TE differentiation.

Using our mESCs model, we demonstrate that a TE lineage factor, Tfap2c, can be directly regulated by LIF/Stat3 signaling. Several pieces of evidence indicate that LIF/Stat3 signaling is involved in TE development during the implantation. Blastocyst TE gives rise to the mammalian placenta, which play a significant role during implantation and gestation. In vivo, expression of LIF is essential for the mammalian endometrium during blastocyst implantation (Stewart et al., 1992), and LIF receptor–null mutant mice show abnormal placental architecture and result in prenatal death (Ware et al., 1995). In addition, Stat3 activity is reported to be necessary for trophoblast differentiation during the implantation process (Fitzgerald et al., 2005; Fitzgerald et al., 2008; Poehlmann et al., 2005). Additionally, enhanced and prolonged Stat3 activity resulting from a lack of its negative regulator, SOCS3, promotes differentiation of trophoblast stem cells (Takahashi et al., 2008). These physiological results echo our finding that TE differentiation can be regulated by the LIF/Stat3 pathway, implying that Tfap2c might also be a key component in LIF/Stat3 mediated TE and placenta development.

The degree of Stat3 activation by extrinsic factors might be subtly different among ESCs from different species, and these subtle differences might account for the failure to establish authentic ESCs from non-rodent species under the LIF condition. This hypothesis is supported by our finding that artificially elevated Stat3 signaling supports self-renewal of ESCs derived from the derivation-refractory B6 mouse strain as well the rat (Li et al., 2008). By studying the role of LIF/Stat3 signaling in isolation and in context within mESCs, we might gain insights into why LIF is not sufficient to promote self-renewal in ESCs from other species, which would support a universal latent mammalian self-renewal mechanism. The results presented in this study suggest that activation of LIF/Stat3 signaling in mESCs can induce the expression of pluripotency-related genes as well as differentiation-inducing genes such as Tfap2c and Cdx2. Rat inner cell mass cells cultured in LIF alone differentiate mainly into Cdx2 positive trophectoderm lineage (Buehr et al., 2003). We speculate that rat ESCs can be maintained by LIF if the induction of differentiation-related genes by LIF/Stat3 signaling is suppressed. LIF/Stat3 signaling may also be sufficient to maintain self-renewal of ESCs from other species if the induction of differentiation-related genes is specifically blocked. Therefore, we anticipate that the differentiation-related genes upregulated by Stat3 must also be finely controlled to ensure ESC self-renewal and that controlled modulation of Stat3 function can facilitate the establishment of authentic non-rodent ESCs.

## MATERIALS AND METHODS

### Cell culture and AP staining

B6 ESCs were routinely maintained on feeders in DMEM medium (Gibco) containing 10% FBS (HyClone), 1 mM Sodium Pyruvate (Gibco), 100 µM Non-Essential Amino Acids (Gibco), 2 mM GLUTAMAX (Gibco), 1 µM β-mercaptoethanol (a formulation hereinafter referred to as “mESC medium”), and 1000 U/ml LIF (Stemgent). The concentration of GCSF used in this study was 50 ng/ml unless specifically indicated. 46C ESCs were routinely maintained on gelatin-coated plates in mESC medium. Trophoblast stem cell medium was prepared according to the previous report (Tanaka et al., 1998). Alkaline phosphatase (AP) staining was performed with an alkaline phosphatase kit (Sigma) according to the manufacturer's instructions.

### Plasmid construction and gene transfection

Stat3 and grgp130-Y118F transgenes were cloned into the pCAG-IRES-IP expression vector or the pSIN-EF2 lentiviral vector. The details on how to generate grgp130-Y118F transgene have been described in a previous study (Burdon et al., 1999). pCAG-Stat3 and pCAG-grgp130-Y118F plasmids were introduced into ESCs using Lipofectamine LTX and Plus reagent (Invitrogen). 293T cells were cultured in DMEM medium containing 10% FBS for lentiviral packaging. 5 µg pSPAX2 (Addgene), 3 µg pVSVG (Addgene) and 8 µg of pSIN-EF2-Sta3 or pSIN-EF2-grgp130-Y118F were transfected into 293T cells using Lipofectamine LTX and Plus reagent (Invitrogen). Virus-containing supernatant was collected and filtered 48 hours after transfection. For lentiviral infection, mESCs were seeded at 10^5^ cells per well into a 24-well plate and cultured in 600 µl of medium per well composed of 300 µl filtered virus-containing supernatant and 300 µl mESC medium with 4 µg/ml Polybrene (Millipore).

### qRT-PCR

Total RNA was extracted with the Quick-RNA MiniPrep Kit (Zymo). cDNA was synthesized with 0.5 µg of total RNA, using the QuantiTech Rev. Transcription Kit (Qiagen). qRT-PCR was performed with Power SYBR Green PCR Master Mix (Applied Biosystems) according to the manufacturer's instructions. Signals were detected with an ABI7900HT real-time PCR System (Applied Biosystems). The relative expression level was determined by the 2-ΔCT method and normalized against *Gapdh*. The primers used for qRT-PCR are listed in supplementary material Table S1.

### Gene knockdown

shRNA-expressing plasmids were generated according to Addgene PLKO.1 protocol. Certain genes which have no validated shRNA were knocked down by introduction of multiple shRNA sequences into cells to achieve better efficiency. The target-specific shRNA sequences used in this study are as follows: Control shRNA: AATTCTCCGAACGTGTCACGT; Cdx2 shRNA: GGACAGAAGATGAGTGGAATT; Gata3 shRNA#1: GCCTGCGGACTCTACCATAA A; Gata3 shRNA#2: ATTGCTGAACATTGCATATAA; Gata3 shRNA#3: CAGTTGTTTGATG CATTTAAA; Tead4 shRNA#1: CCGCCAAATCTATGACAAGTT; Tead4 shRNA#2: GC TGAAACACTTACCCGAGAA; Tead4 shRNA#3: CCCTCT CTGTGAGTACATGAT; Tfap2c shRNA: AGCCGCTCTGCAAGTCTAATA. After lentiviral infection, the cells were incubated with 1 µg/ml puromycin, 10 µg/ml Blasticidin S deaminase, or 100 µg/ml hygromycin for 48–72 hours. The surviving ESCs were pulled together to examine knockdown efficiency and to investigate their roles in GCSF induced TE differentiation.

### Immunostaining

Immunostaining was performed according to a standard protocol. Primary antibodies used were the following: Cdx2 (3977S, Cell Signaling, 1:200). Alexa Flour fluorescent secondary antibodies (Invitrogen) were used at a 1:2000 dilution. Nuclei were visualized with DAPI.

## Supplementary Material

Supplementary Material
