# Supplementary material for: Stat3 signaling regulates embryonic stem cell fate in a dose-dependent manner
Source: Biol Open. 2014 Sep 19;3(10):958–65. doi: 10.1242/bio.20149514 (PMC4197444; doi:10.1242/bio.20149514)
Supplement: Supplementary Material [file supp_3_10_958__index.html]

Stat3 signaling regulates embryonic stem cell fate in a dose-dependent manner — Supplementary Material 

# Stat3 signaling regulates embryonic stem cell fate in a dose-dependent manner

## bio.20149514 Supplementary Material

**Files in this Data Supplement:**

- Supplementary Material - Chih-I Tai et al. doi: 10.1242/bio.20149514
