# Supplementary material for: Stat3 signaling regulates embryonic stem cell fate in a dose-dependent manner
Source: Biol Open. 2014 Sep 19;3(10):958–65. doi: 10.1242/bio.20149514 (PMC4197444; doi:10.1242/bio.20149514)
Supplement: Supplementary Material [file supp_bio.20149514_bio.20149514-s1.pdf]

Supplementary Material

Chih-I Tai et al. doi: 10.1242/bio.20149514

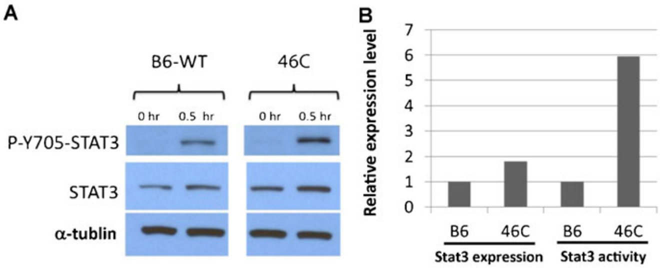

Fig. S1. Total and phosphorylated Stat3 levels in B6 and 46C mESCs. (A) Western blot analysis of phospho-Stat3 (Tyr705) and total Stat3 expression levels in B6-WT and 46C mESCs treated with or without LIF for 30 mins. B6-WT and 46C mESCs were starved in serum free medium for overnight before the treatment. (B) Relative expression levels of total and phosphorylated Stat3 in B6-WT and 46C mESCs treated with LIF for 30 mins. The Stat3 expression levels were normalized to α-tublin.

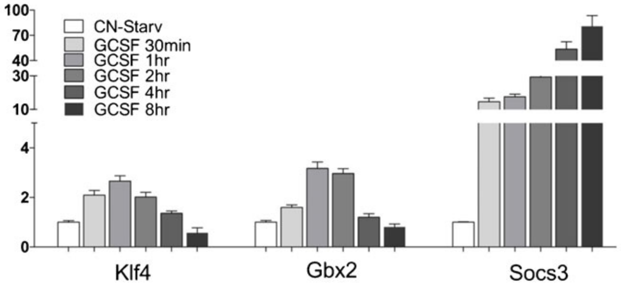

Fig. S3. qPCR analysis of the expression of Stat3 target genes in B6-S3Y118F mESCs treated with GCSF for the indicated times.

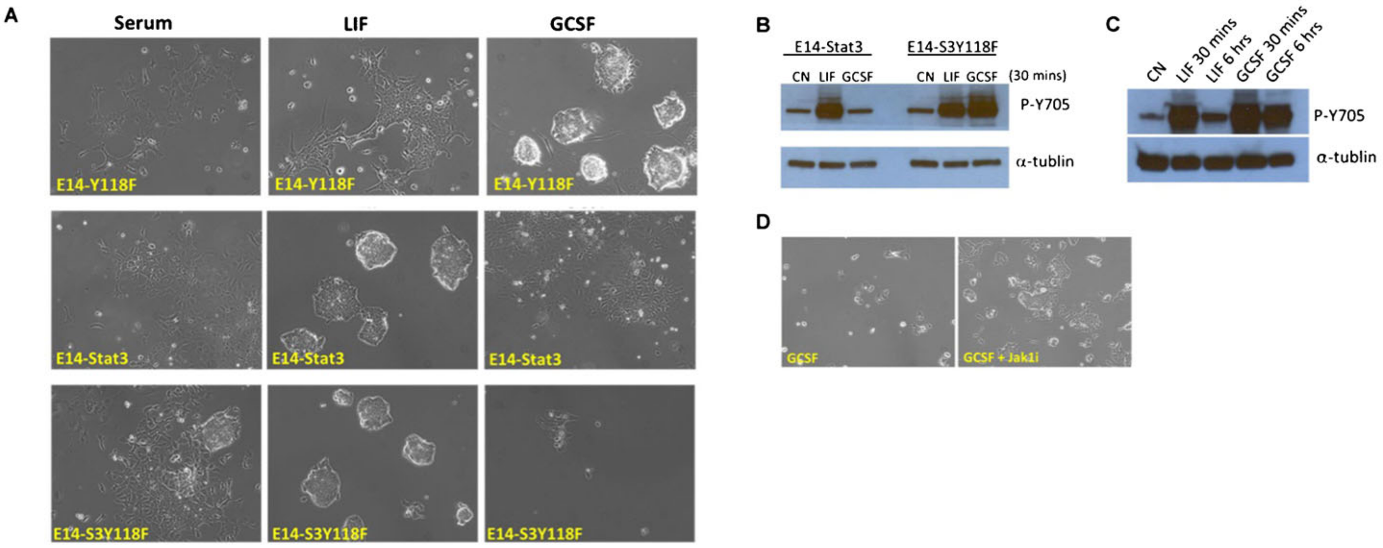

Fig. S2. Hyperactivation of Stat3 induces differentiation of E14-S3Y118F mESCs. (A) Phase contrast images of E14-Y118F, E14-Stat3, and E14-S3Y118F mESCs cultured in mESC medium only or mESC medium supplemented with LIF or GCSF. Images were taken 6 days after plating. (B) Western blot analysis of phospho-Stat3 (Tyr705) levels in E14-Stat3 and E14-S3Y118F mESCs treated with LIF or GCSF for 30 mins. Both E14-Stat3 and E14-S3Y118F mESCs were starved in the serum free medium for overnight before the treatment. (C) Western blot analysis of phospho-Stat3 (Tyr705) levels in E14-S3Y118F ESCs treated with LIF or GCSF for 30 mins or 6 hours. The mESCs were starved in the serum free medium for overnight before the treatment. (D) Phase contrast images of E14-S3Y118F ESCs cultured in GCSF or GCSF plus JAK1i for 3 days.

Biology Open (2014) 000, 1–8 doi:10.1242/bio.20149514

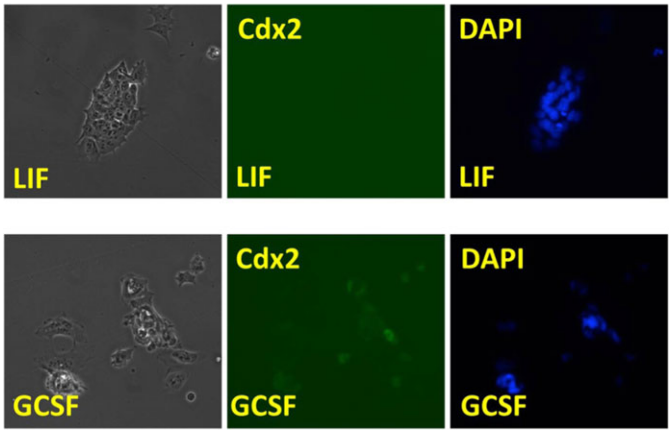

Fig. S4. Immunofluorescence staining of Cdx2 in B6-S3Y118F mESCs cultured in the presence of LIF or GCSF for 48 h.

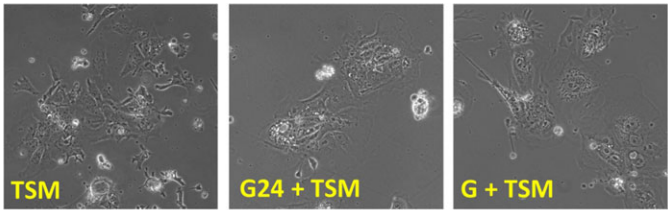

Fig. S5. GCSF induces B6-S3Y118F mESC differentiation towards terminally differentiated TE cells. Left panel: phase contrast image of B6-S3Y118F mESCs cultured in trophoblast stem cell medium (TSM) for 5 days; middle panel: phase contrast image of B6-S3Y118F mESCs treated with GCSF for 24 h and then cultured in TSM for 5 days; right panel: phase contrast image of B6-S3Y118F mESCs cultured in TSM supplemented with GCSF for 5 days.

Table S1. The list of primers used for qRT-PCR analysis

Symbol	Primer sequence for RT-PCR (5′→3′)	Symbol	Primer sequence for RT-PCR (5′→3′)
Gbx2	F: TCGCTGCTCGCTTTCTCT	Nestin	F: CTCGAGCAGGAAGTGGTAGG
	R: GGGTCATCTTCCACCTTTGA		R: TTGGGACCAGGGACTGTTAG
Klf4	F: CGAACTCACACAGGCGAGAA	Sox1	F: GGCCGAGTGGAAGGTCATGT
	R: CGGAGCGGGCGAATTT		R: TCCGGGTGTTCCTTCATGTG
Esrrb	F: AACAGCCCCTACCTGAACCT	Mixl1	F: TTGAATTGAACCCTGTTGTCCC
	R: CTCATCTGGTCCCCAAGTGT		R: GAAACCCGTTCTCCCATCCACC
Oct4	F: GAAGCAGAAGAGGATCACCTTG	T	F: CCGGTGCTGAAGGTAAATGT
	R: TTCTTAAGGCTGAGCTGCAAG		R: CCTCCATTGAGCTTGTTGGT
Sox2	F: ATGGGCTCTGTGGTCAAGTC	Eomes	F: GGCAAAGCGGACAATAACAT
	R: CCCTCCCAATTCCCTTGTAT		R: AGCCTCGGTTGGTATTTGTG
Gata4	F: TCTCACTATGGGCACAGCAG	Cdx2	F: ACCGGAATTGTTTGCTGCTGT
	R: GCGATGTCTGAGTGACAGGA		R: TCCCGACTTCCCTTCACCAT
Gata6	F: TCCTCCCCTGCCGAAGTC	Dlx3	F: TACTCGCCCAAGTCGGAATA
	R: AGGGCCAGAGCACACCAA		R: AGTAGATCGTTCGCGGCTTT
FoxA2	F: CCTCAAGGGAGCAGTCTCAC	Esx1	F: GAGCTGGAGGCCTTTTTCCA
	R: TTTCTCCTGGTCCGGTACAC		R: ACACCCACAGGGGGACTCAT
Sox17	F: AGCCATTTCCTCCGTGGTGT	Gata3	F: GGGCTACGGTGCAGAGGTAT
	R: AACACTGCTTCTGGCCCTCAG		R: TGGATGGACGTCTTGGAGAA
Psx1	F: CGATGGATGGGTGTGGATGA	Tfap2c	F: ATCCCTCACCTCTCCTCTCC
	R: TGACAGGGCTGGCACTCAAG		R: CCAGATGCGAGTAATGGTCGG
Gapdh	F: TGTGAGGGAGATGCTCAGTG		
	R: TGTTCCTACCCCCAATGTGT		
